# Crystallographic and spectroscopic character­ization of 4-nitro-2-(tri­fluoro­meth­yl)benzoic acid and 4-nitro-3-(tri­fluoro­meth­yl)benzoic acid

**DOI:** 10.1107/S2056989019003979

**Published:** 2019-03-29

**Authors:** George L. Diehl III, Lisa Je, Joseph M. Tanski

**Affiliations:** a Department of Chemistry, Vassar College, Poughkeepsie, NY 12604, USA

**Keywords:** crystal structure, hydrogen bonding, benzoic acid derivatives, tri­fluoro­methyl group

## Abstract

The title compounds, both C_8_H_4_F_3_NO_4_, represent two isomers of nitro tri­fluoro­methyl benzoic acid. The compounds each contain a nitro functionality *para* to the carb­oxy­lic acid group, with the tri­fluoro­methyl substituent *ortho* to the acid group in the 2-isomer and *ortho* to the nitro group in the 3-isomer. The regiochemistry with respect to the tri­fluoro­methyl group results in steric inter­actions that rotate the carb­oxy­lic acid group or the nitro group out of the aromatic plane in the 2- and 3-isomer, respectively. Each mol­ecule engages in inter­molecular hydrogen bonding, forming head-to-tail dimers with graph-set notation *R*_2_^2^(8) and donor–acceptor hydrogen-bonding distances of 2.7042 (14) Å in the 2-isomer and 2.6337 (16) in the 3-isomer. Recrystallization attempts did not yield untwinned crystals.

## Abstract 2

The title compounds, two isomers of nitro tri­fluoro­methyl benzoic acid, each contain a nitro functionality *para* to the carb­oxy­lic acid group, with the tri­fluoro­methyl substituent *ortho* to the acid group in the 2-isomer and *ortho* to the nitro group in the 3-isomer. The regiochemistry with respect to the tri­fluoro­methyl group results in steric inter­actions that rotate the carb­oxy­lic acid group or the nitro group out of the aromatic plane in the 2- and 3-isomer, respectively.

## Chemical context   

The title compounds, 4-nitro-2-(tri­fluoro­meth­yl)benzoic acid (I) and 4-nitro-3-(tri­fluoro­meth­yl)benzoic acid (II), are tri-substituted aromatic compounds featuring a carb­oxy­lic acid, a nitro group and a tri­fluoro­methyl group. Although all ten isomers of nitro tri­fluoro­methyl benzoic acid are available commercially, none of their crystal structures have been reported. 4-Nitro-2-(tri­fluoro­meth­yl)benzoic acid (I) may be synthesized from 2-(tri­fluoro­meth­yl)benzoic acid by treating it with concentrated sulfuric acid, stirring, and adding fuming nitric acid dropwise (Kompella *et al.*, 2017 ▸). 4-Nitro-2-(tri­fluoro­meth­yl)benzoic acid (I) has been used in the syntheses of potential pharmaceuticals, for example in anti-tumor pyridinone (Cheung *et al.*, 2017 ▸) and urea derivatives (Nishio *et al.*, 2017 ▸). 4-Nitro-3-(tri­fluoro­meth­yl)benzoic acid (II) was first reported in 1951 after being prepared from the corresponding nitrile (Caldwell & Sayin, 1951 ▸). The compound has recently been used for the synthesis of glutamate receptor antagonists (Selvam *et al.*, 2018 ▸) that have potential as therapies for diseases such as Parkinson’s.
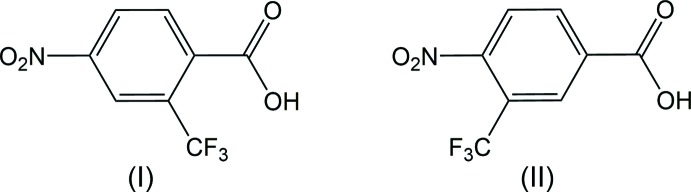


## Structural commentary   

**Figure 1:**
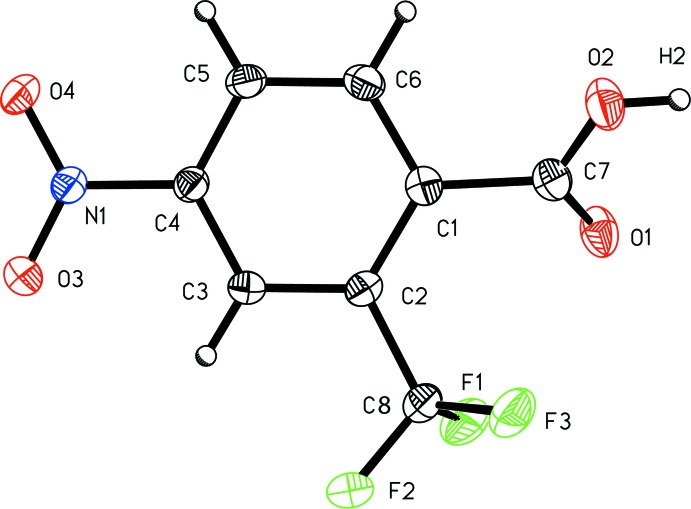
A view of 4-nitro-2-(tri­fluoro­meth­yl)benzoic acid (I) with the atom-numbering scheme. Displacement ellipsoids are shown at the 50% probability level.

**Figure 2:**
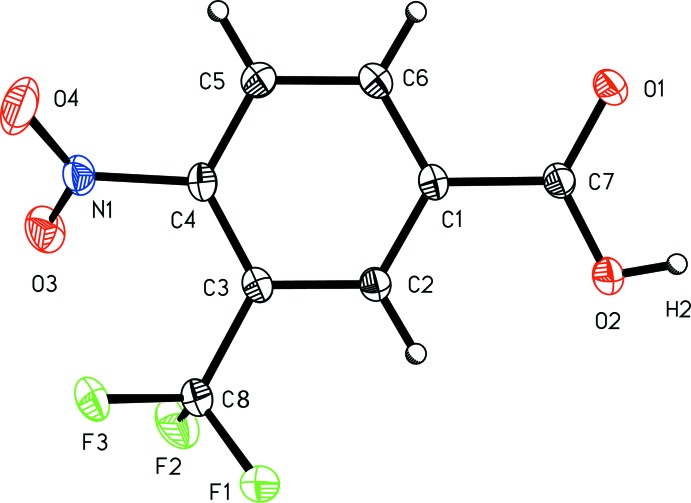
A view of 4-nitro-3-(tri­fluoro­meth­yl)benzoic acid (II) with the atom-numbering scheme. Displacement ellipsoids are shown at the 50% probability level.

4-Nitro-2-(tri­fluoro­meth­yl)benzoic acid, (I) (Fig. 1 ▸), and 4-nitro-3-(tri­fluoro­meth­yl)benzoic acid, (II) (Fig. 2 ▸), exhibit similar metrical parameters. The aromatic nitro bond length C4—N1 of 1.4718 (16) Å in (I) and 1.4751 (19) in (II) are similar, as are the aromatic tri­fluoro­methyl bond lengths C2—C8 of 1.5114 (17) Å in (I) and C3—C8 of 1.508 (2) Å in (II). The nitro N—O distances lie between 1.2154 (19) and 1.2271 (14) Å; average 1.224 (6) Å. Whereas the carb­oxy­lic acid group in (I) is not significantly disordered, with an O1—C7 carbonyl bond length of 1.219 (2) Å and an O2—C7 acid bond length of 1.3139 (16) Å, the carb­oxy­lic acid group in (II) exhibits some twofold disorder, with an O1—C7 bond length of 1.2528 (18) Å and O2—C7 acid bond length of 1.281 (2) Å.

**Figure 3:**
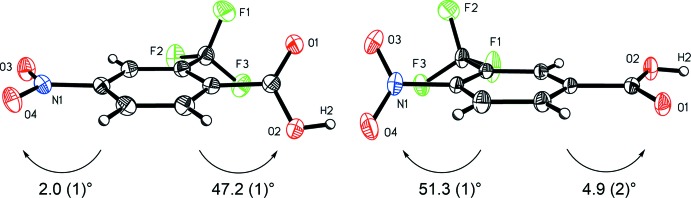
Side-by-side views of 4-nitro-2-(tri­fluoro­meth­yl)benzoic acid (I) (left) and 4-nitro-3-(tri­fluoro­meth­yl)benzoic acid (II) indicating the rotation of the carboxyl and nitro groups out of the mean plane of the aromatic ring.

A notable difference in the mol­ecular structures of the title compounds is the influence of the tri­fluoro­methyl substituent on the co-planarity of the carb­oxy­lic acid and nitro groups with the aromatic ring plane (Fig. 3 ▸). In 4-nitro-2-(tri­fluoro­meth­yl)benzoic acid (I), the tri­fluoro­methyl group *ortho* to the carb­oxy­lic acid moiety rotates it out of the plane of the aromatic ring, with a plane-to-plane angle of 47.2 (1)°, whereas the nitro group is almost co-planar with the aromatic ring, with an angle of 2.0 (1)°. Conversely, in 4-nitro-3-(tri­fluoro­meth­yl)benzoic acid (II), the tri­fluoro­methyl group *ortho* to the nitro moiety rotates it out of the plane of the aromatic ring, with a plane-to-plane angle of 51.3 (1)°, whereas the carb­oxy­lic acid group is closer to co-planar with the aromatic ring, with an angle of 4.9 (2)°.

## Supra­molecular features   

**Figure 4:**
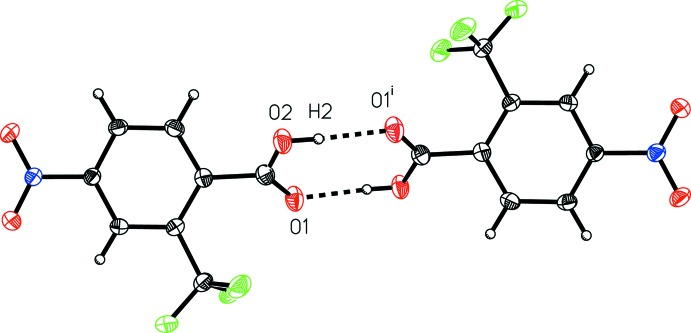
A view of the inter­molecular hydrogen bonding in 4-nitro-2-(tri­fluoro­meth­yl)benzoic acid (I). [Symmetry code: (i) −*x* + 1, −*y* + 1, −*z*.]

**Figure 5:**
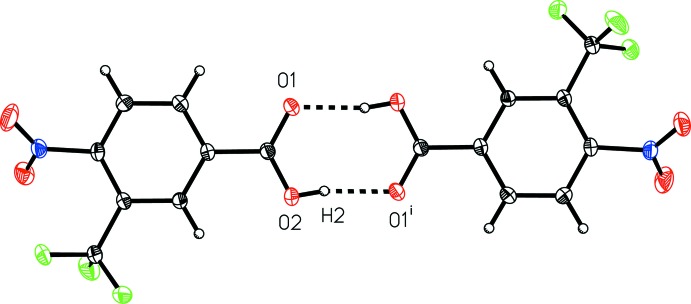
A view of the inter­molecular hydrogen bonding in 4-nitro-3-(tri­fluoro­meth­yl)benzoic acid (II). [Symmetry code: (i) −*x*, −*y* + 1, −*z*.]

The mol­ecules of the title compounds pack together in the solid state with hydrogen bonding between the carb­oxy­lic acid hydrogen atom and the carbonyl oxygen atom of the symmetry-related carboxyl group in a neighboring mol­ecule, forming a dimer with graph-set notation 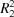
(8). This centrosymmetric pairwise hydrogen-bonding dimer formation results in short hydrogen-bonding distances of 2.7042 (14) Å in (I) (Fig. 4 ▸, Table 1 ▸) and 2.6337 (16) in (II) (Fig. 5 ▸, Table 2 ▸).

**Figure 6:**
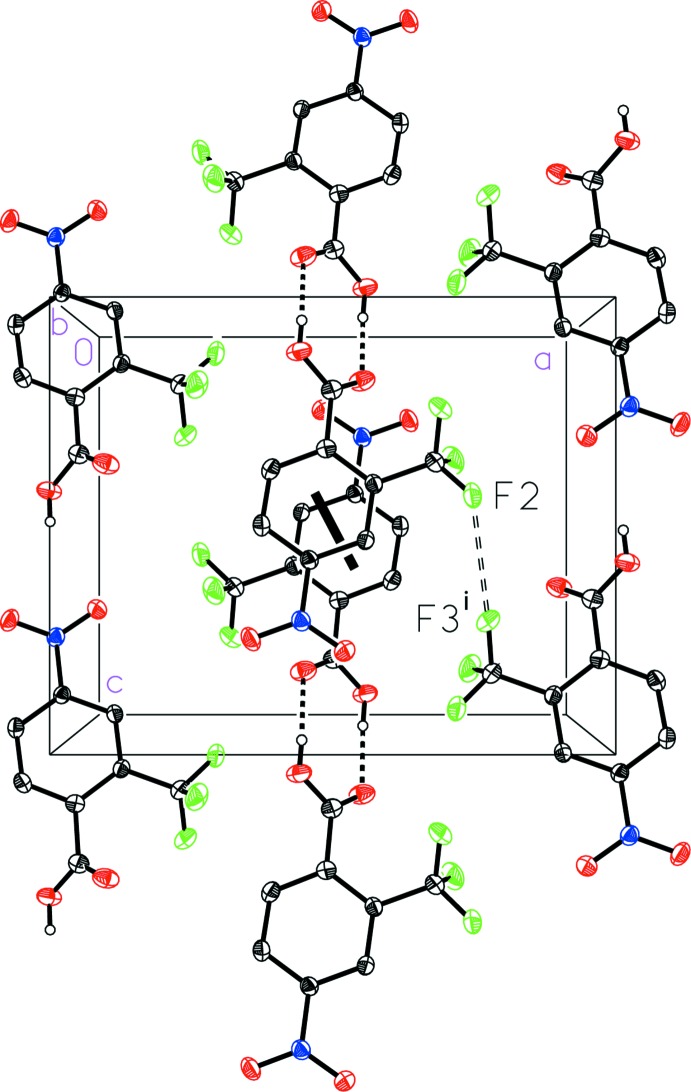
A view of the packing in 4-nitro-2-(tri­fluoro­meth­yl)benzoic acid (I) with a double-dashed line indicating the F⋯F inter­action and a thick solid line indicating a centroid-to-centroid inter­action. [Symmetry code: (i) −*x* + 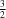, *y*, *z* + 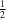.]

**Figure 7:**
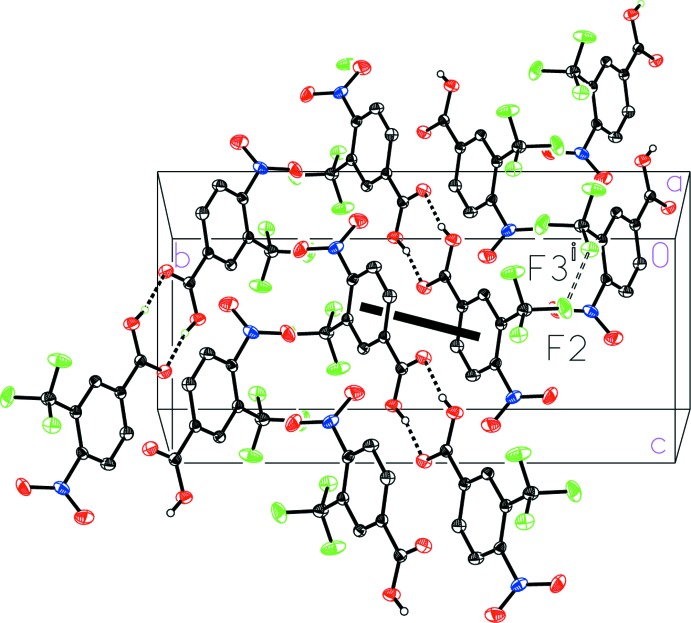
A view of the packing in 4-nitro-3-(tri­fluoro­meth­yl)benzoic acid (II) with a double-dashed line indicating the F⋯F inter­action and a thick solid line indicating a centroid-to-centroid inter­action. [Symmetry code: (i) −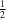 + *x*, 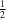 − *y*, −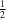 + *z*.]

The mol­ecular packing in the unit cell of 4-nitro-2-(tri­fluoro­meth­yl)benzoic acid (I) (Fig. 6 ▸) reveals a dimerized face-to-face geometrical arrangement of the aromatic rings related by inversion, with a ring centroid-to-centroid distance of 3.907 (1) Å, a centroid-to-plane distance of 3.820 (1) Å, and a ring-offset slippage of 0.822 (2) Å. An inter­molecular fluorine–fluorine inter­action is also observed with a length of 2.927 (1) Å that is similar to the sum of the van der Waals radii (2.94 Å; Bondi, 1964 ▸). The hydrogen bonded dimers of 4-nitro-3-(tri­fluoro­meth­yl)benzoic acid (II) pack together in a similar way, but with a longer fluorine–fluorine contact [2.975 (2) Å] and a highly offset face-to-face geometric arrangement of the aromatic rings characterized by a large ring-offset slippage of 1.733 (2) Å such that the aromatic rings are barely overlapped (Fig. 7 ▸).

## Database survey   

The Cambridge Structural Database (Groom *et al.*, 2016 ▸) contains no isomers of nitro tri­fluoro­methyl benzoic acid. A related derivative of 4-nitro-3-(tri­fluoro­meth­yl)benzoic acid (II) is 3-methyl-4-nitro­benzoic acid (TOYGIZ), which exhibits a similar hydrogen-bonding motif and hydrogen-bonding distance of 2.617 Å (Saha *et al.*, 2015 ▸). As with (II), the methyl group *ortho* to the nitro moiety in TOYGIZ rotates it out of the plane of the aromatic ring whereas the carb­oxy­lic acid group is closer to co-planar with the aromatic ring.

## Synthesis and crystallization   

4-Nitro-2-(tri­fluoro­meth­yl)benzoic acid (I) (97%) was purchased from Alfa Aesar and 4-nitro-3-(tri­fluoro­meth­yl)benzoic acid (II) (97%) were purchased from Aldrich Chemical Company. (I) was recrystallized from tetra­hydro­furan and (II) was used as received.

## Refinement   

Crystal data, data collection and structure refinement details are summarized in Table 3 ▸. All non-hydrogen atoms were refined anisotropically. Hydrogen atoms on carbon were included in calculated positions and refined using a riding model with C—H = 0.95 and *U*_iso_(H) = 1.2*U*_eq_(C) of the aryl C-atoms the hydrogens are riding on. The positions of the carb­oxy­lic acid hydrogen atoms were found in the difference map and the atoms refined semi-freely using a distance restraint *d*(O—H) = 0.84 Å, and *U*_iso_(H) = 1.2*U*_eq_(O). 4-Nitro-3-(tri­fluoro­meth­yl)benzoic acid (II) was found to be multiply non-merohedrally twinned. Recrystallization attempts did not yield untwinned crystals. Three components were integrated with *SAINT* using the multiple-component orientation matrix produced by *CELL_NOW* (Sheldrick, 2003 ▸), and the data were absorption corrected and scaled with *TWINABS* (Sheldrick, 2008*a* ▸). The initial solution was found and refined with merged and roughly detwinned HKLF 4 format data before final refinement against HKLF5 format data constructed from all observations involving domain 1 only. The twin ratio (*SHELXL* BASF parameters) refined to 0.0961 (3) and 0.0326 (2).

## Analytical data   

(I) ^1^H NMR (Bruker Avance III HD 400 MHz, DMSO *d*_6_): δ 8.07 (*d*, 1 H, C_ar­yl_ *H*, *J*_ortho_ = 8.4 Hz), 8.50 (*d*, 1 H, C_ar­yl_ *H*, *J*_meta_ = 2.2 Hz), 8.56 (*dd*, 1 H, C_ar­yl_ *H*, *J*_ortho_ = 8.4 Hz, *J*_meta_ = 2.2 Hz), 14.28 (*br s*, 1 H, O*H*). ^13^C NMR (^13^C{^1^H}, 100.6 MHz, DMSO *d*_6_): δ 121.76 (*q*, *C*_ar­yl_H, *J*_C-F_ = 5.4 Hz), 122.31 (*q*, *C*F_3_, *J*_C-F_ = 274 Hz), 127.20 (*q*, *C*_ar­yl_CF_3_, *J*_C-F_ = 33.5 Hz), 127.64 (*s*, *C*_ar­yl_H), 131.35 (*s*, *C*_ar­yl_H), 137.86 (*s*, *C*_ar­yl_COOH), 148.27 (*s*, *C*_ar­yl_NO_2_), 166.44 (*s*, *C*OOH). IR (Thermo Nicolet iS50, ATR, cm^−1^): 3133 (*s br*, O—H *str*), 3096 (*s*, C_ar­yl_-H *str*), 2922 (*s*), 2660 (*m*), 2531 (*m*), 1723 (*s*, C=O *str*), 1618 (*s*), 1540 (*s*), 1498 (*m*), 1407 (*s*), 1357 (*s*), 1317 (*s*), 1294 (*s*), 1268 (*s*), 1177 (*m*), 1153 (*s*), 1115 (*s*), 1048 (*s*), 920 (*s*), 899 (*m*), 861 (*m*), 803 (*s*), 769 (*w*), 742 (*m*), 700 (*m*), 656 (*m*), 563 (*m*), 503 (*m*). GC–MS (Agilent Technologies 7890A GC/5975C MS): *M* ^+^ = 249 amu, corres­ponding to the methyl ester of (I), prepared from the parent carb­oxy­lic acid using a literature procedure (Di Raddo, 1993 ▸).

(II) ^1^H NMR (Bruker Avance III HD 400 MHz, DMSO *d*_6_): δ 8.28 (*d*, 1H, C_ar­yl_ *H*, *J*_ortho_ = 8.4 Hz), 8.36 (*d*, 1H, C_ar­yl_ *H*, *J*_meta_ = 1.6 Hz), 8.43 (*dd*, 1 H, C_ar­yl_ *H*, *J*_ortho_ = 8.0 Hz, *J*_meta_ = 1.8 Hz), 14.06 (*br s*, 1H, O*H*). ^13^C NMR (^13^C{^1^H}, 100.6 MHz, DMSO *d*_6_): δ 121.53 (*q*, *C_ar­yl_*CF_3_, *J*_C-F_ = 33.9 Hz), 121.64 (*q*, *C*F_3_, *J*_C-F_ = 273 Hz), 126.0 (*s*, *C_ar­yl_*H), 128.30 (*q*, *C_ar­yl_*H, *J*_C-F_ = 5.2 Hz), 135.02 (*s*, C_ar­yl_ *H*), 135.13 (*s*, *C*_ar­yl_COOH), 149.38 (*s*, *C*_ar­yl_ NO_2_), 164.48 (*s*, *C*OOH). ^19^F NMR (^19^F{^1^H}, 376.5 MHz, DMSO *d*_6_): −59.24 (*s*, 3F, C*F_3_*). IR (Thermo Scientific iS50, ATR, cm^−1^): 3104 (*m br*, O-H *str*), 3067 (*m*, C_ar­yl_-H *str*), 2848 (*m*), 2646 (*m*), 2575 (*m*), 1700 (*s*, C=O *str*), 1618 (*m*), 1598 (*m*), 1548 (*s*), 1438 (*m*), 1409 (*m*), 1363 (*m*), 1313 (*m*), 1267 (*s*) 1176 (*m*), 1163 (*s*), 1140 (*s*), 1125 (*s*), 1049 (*m*), 912 (*m*), 889 (*m*), 827 (*m*), 779 (*m*), 766 (*m*), 747 (*m*), 721 (*w*), 702 (*m*), 654 (*m*), 616 (*w*), 545 (*m*), 506 (*m*), 419 (*m*). GC–MS (Agilent Technologies 7890A GC/5975C MS): *M* ^+^ = 249 amu, corresponding to the methyl ester of (II), prepared from the parent carb­oxy­lic acid using a literature procedure (Raddo, 1993 ▸).

## Supplementary Material

Crystal structure: contains datablock(s) global, I, II. DOI: 10.1107/S2056989019003979/pk2616sup1.cif

Structure factors: contains datablock(s) I. DOI: 10.1107/S2056989019003979/pk2616Isup2.hkl

Structure factors: contains datablock(s) II. DOI: 10.1107/S2056989019003979/pk2616IIsup3.hkl

Supporting information file. DOI: 10.1107/S2056989019003979/pk2616Isup4.cml

Supporting information file. DOI: 10.1107/S2056989019003979/pk2616IIsup5.cml

CCDC references: 1905077, 1905076

Additional supporting information:  crystallographic information; 3D view; checkCIF report

## Figures and Tables

**Table 1:** Hydrogen-bond geometry (Å, °) for (I)

*D*—H⋯*A*	*D*—H	H⋯*A*	*D*⋯*A*	*D*—H⋯*A*
O2—H2⋯O1^i^	0.85 (1)	1.86 (2)	2.7042 (14)	175 (2)

Symmetry code: (i) 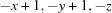.

**Table 2:** Hydrogen-bond geometry (Å, °) for (II)

*D*—H⋯*A*	*D*—H	H⋯*A*	*D*⋯*A*	*D*—H⋯*A*
O2—H2⋯O1^i^	0.83 (2)	1.82 (2)	2.6337 (16)	168 (2)

Symmetry code: (i) 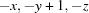.

**Table 3:** Experimental details

	(I)	(II)
Crystal data		
Chemical formula	C_8_H_4_F_3_NO_4_	C_8_H_4_F_3_NO_4_
*M*_r_	235.12	235.12
Crystal system, space group	Orthorhombic, *P* *c* *c* *n*	Monoclinic, *P*2_1_/*n*
Temperature (K)	125	125
*a*, *b*, *c* (Å)	12.1612 (17), 14.847 (2), 9.8265 (14)	6.8986 (8), 17.240 (2), 7.6912 (9)
α, β, γ (°)	90, 90, 90	90, 107.685 (2), 90
*V* (Å^3^)	1774.2 (4)	871.50 (18)
*Z*	8	4
Radiation type	Mo *K*α	Mo *K*α
μ (mm^−1^)	0.18	0.18
Crystal size (mm)	0.24 × 0.24 × 0.15	0.30 × 0.20 × 0.10

Data collection		
Diffractometer	Bruker APEXII CCD	Bruker APEXII CCD
Absorption correction	Multi-scan (*SADABS*; Bruker, 2013 ▸)	Multi-scan (*TWINABS*; Sheldrick, 2008*a* ▸)
*T*_min_, *T*_max_	0.86, 0.97	0.89, 0.98
No. of measured, independent and observed [*I* > 2σ(*I*)] reflections	40170, 2727, 2064	4385, 2665, 2116
*R*_int_	0.050	0.071
(sin θ/λ)_max_ (Å^−1^)	0.716	0.715

Refinement		
*R*[*F* ^2^ > 2σ(*F* ^2^)], *wR*(*F* ^2^), *S*	0.039, 0.109, 1.04	0.048, 0.150, 1.05
No. of reflections	2727	2873
No. of parameters	148	150
No. of restraints	1	1
H-atom treatment	H atoms treated by a mixture of independent and constrained refinement	H atoms treated by a mixture of independent and constrained refinement
Δρ_max_, Δρ_min_ (e Å^−3^)	0.46, −0.24	0.50, −0.36

Computer programs: *APEX2* and *SAINT* (Bruker, 2013 ▸), *SHELXT2014* (Sheldrick, 2015*a* ▸), *SHELXL2016* (Sheldrick, 2015*b* ▸), *SHELXTL* (Sheldrick, 2008*b* ▸), *OLEX2* (Dolomanov *et al.*, 2009 ▸) and *Mercury* (Macrae *et al.*, 2008 ▸).

## supplementary crystallographic information

### 4-Nitro-2-(trifluoromethyl)benzoic acid (I) Crystal data

**Table:**

C_8_H_4_F_3_NO_4_	*D*_x_ = 1.760 Mg m^−^^3^
*M**_r_* = 235.12	Mo *K*α radiation, λ = 0.71073 Å
Orthorhombic, *P**c**c**n*	Cell parameters from 7558 reflections
*a* = 12.1612 (17) Å	θ = 2.2–30.2°
*b* = 14.847 (2) Å	µ = 0.18 mm^−^^1^
*c* = 9.8265 (14) Å	*T* = 125 K
*V* = 1774.2 (4) Å^3^	Block, colourless
*Z* = 8	0.24 × 0.24 × 0.15 mm
*F*(000) = 944	

### 4-Nitro-2-(trifluoromethyl)benzoic acid (I) Data collection

**Table:**

Bruker APEXII CCD diffractometer	2727 independent reflections
Radiation source: fine-focus sealed tube	2064 reflections with *I* > 2σ(*I*)
Graphite monochromator	*R*_int_ = 0.050
Detector resolution: 8.3333 pixels mm^-1^	θ_max_ = 30.6°, θ_min_ = 2.2°
φ and ω scans	*h* = −17→17
Absorption correction: multi-scan (SADABS; Bruker, 2013)	*k* = −21→21
*T*_min_ = 0.86, *T*_max_ = 0.97	*l* = −14→14
40170 measured reflections	

### 4-Nitro-2-(trifluoromethyl)benzoic acid (I) Refinement

**Table:**

Refinement on *F*^2^	1 restraint
Least-squares matrix: full	Hydrogen site location: mixed
*R*[*F*^2^ > 2σ(*F*^2^)] = 0.039	H atoms treated by a mixture of independent and constrained refinement
*wR*(*F*^2^) = 0.109	*w* = 1/[σ^2^(*F*_o_^2^) + (0.0543*P*)^2^ + 0.5851*P*] where *P* = (*F*_o_^2^ + 2*F*_c_^2^)/3
*S* = 1.04	(Δ/σ)_max_ = 0.001
2727 reflections	Δρ_max_ = 0.46 e Å^−^^3^
148 parameters	Δρ_min_ = −0.24 e Å^−^^3^

### 4-Nitro-2-(trifluoromethyl)benzoic acid (I) Special details

**Table:**

Geometry. All esds (except the esd in the dihedral angle between two l.s. planes) are estimated using the full covariance matrix. The cell esds are taken into account individually in the estimation of esds in distances, angles and torsion angles; correlations between esds in cell parameters are only used when they are defined by crystal symmetry. An approximate (isotropic) treatment of cell esds is used for estimating esds involving l.s. planes.

### 4-Nitro-2-(trifluoromethyl)benzoic acid (I) Fractional atomic coordinates and isotropic or equivalent isotropic displacement parameters (Å^2^)

**Table:**

	*x*	*y*	*z*	*U*_iso_*/*U*_eq_	
F1	0.73315 (7)	0.52183 (6)	0.34266 (10)	0.0370 (2)	
F2	0.76500 (7)	0.65169 (7)	0.42859 (9)	0.0367 (2)	
F3	0.70454 (7)	0.64131 (6)	0.22366 (9)	0.0347 (2)	
O1	0.55716 (9)	0.49377 (7)	0.14946 (10)	0.0306 (2)	
O2	0.43545 (9)	0.59970 (7)	0.08655 (10)	0.0289 (2)	
H2	0.4403 (15)	0.5727 (12)	0.0103 (16)	0.035*	
O3	0.52250 (8)	0.69576 (7)	0.79079 (10)	0.0285 (2)	
O4	0.34577 (8)	0.69044 (7)	0.76367 (10)	0.0284 (2)	
N1	0.44022 (9)	0.68191 (7)	0.72167 (11)	0.0211 (2)	
C1	0.48757 (10)	0.59507 (8)	0.31669 (12)	0.0198 (2)	
C2	0.57846 (10)	0.61229 (8)	0.40071 (12)	0.0192 (2)	
C3	0.56260 (10)	0.64170 (8)	0.53324 (13)	0.0193 (2)	
H3A	0.623518	0.65393	0.590881	0.023*	
C4	0.45566 (10)	0.65286 (8)	0.57974 (12)	0.0187 (2)	
C5	0.36455 (10)	0.63879 (8)	0.49877 (13)	0.0213 (2)	
H5A	0.29241	0.648629	0.53275	0.026*	
C6	0.38158 (10)	0.60982 (9)	0.36620 (13)	0.0222 (3)	
H6A	0.320197	0.599895	0.308322	0.027*	
C7	0.49830 (11)	0.55800 (9)	0.17519 (13)	0.0225 (3)	
C8	0.69530 (11)	0.60658 (9)	0.34899 (13)	0.0247 (3)	

### 4-Nitro-2-(trifluoromethyl)benzoic acid (I) Atomic displacement parameters (Å^2^)

**Table:**

	*U*^11^	*U*^22^	*U*^33^	*U*^12^	*U*^13^	*U*^23^
F1	0.0285 (5)	0.0381 (5)	0.0443 (5)	0.0138 (4)	0.0073 (4)	−0.0025 (4)
F2	0.0182 (4)	0.0570 (6)	0.0350 (5)	−0.0067 (4)	0.0034 (3)	−0.0103 (4)
F3	0.0296 (4)	0.0487 (6)	0.0259 (4)	−0.0011 (4)	0.0093 (3)	0.0043 (4)
O1	0.0402 (6)	0.0281 (5)	0.0235 (5)	0.0113 (4)	−0.0049 (4)	−0.0053 (4)
O2	0.0364 (5)	0.0316 (5)	0.0188 (4)	0.0093 (4)	−0.0041 (4)	−0.0024 (4)
O3	0.0230 (5)	0.0384 (6)	0.0241 (5)	−0.0012 (4)	−0.0023 (4)	−0.0077 (4)
O4	0.0206 (4)	0.0340 (5)	0.0307 (5)	0.0012 (4)	0.0075 (4)	−0.0080 (4)
N1	0.0194 (5)	0.0214 (5)	0.0225 (5)	0.0008 (4)	0.0020 (4)	−0.0030 (4)
C1	0.0220 (6)	0.0180 (5)	0.0195 (5)	0.0006 (4)	−0.0006 (4)	0.0002 (4)
C2	0.0170 (5)	0.0202 (6)	0.0203 (6)	0.0015 (4)	0.0021 (4)	0.0011 (4)
C3	0.0160 (5)	0.0213 (6)	0.0206 (5)	0.0003 (4)	−0.0014 (4)	−0.0002 (4)
C4	0.0178 (5)	0.0185 (5)	0.0197 (5)	0.0008 (4)	0.0013 (4)	−0.0011 (4)
C5	0.0158 (5)	0.0223 (6)	0.0259 (6)	0.0008 (4)	0.0002 (4)	−0.0013 (5)
C6	0.0191 (5)	0.0230 (6)	0.0247 (6)	−0.0006 (4)	−0.0042 (5)	−0.0019 (5)
C7	0.0254 (6)	0.0204 (6)	0.0218 (6)	−0.0007 (5)	−0.0014 (5)	−0.0001 (4)
C8	0.0203 (6)	0.0312 (7)	0.0224 (6)	0.0017 (5)	0.0031 (5)	−0.0012 (5)

### 4-Nitro-2-(trifluoromethyl)benzoic acid (I) Geometric parameters (Å, º)

**Table:**

F1—C8	1.3413 (16)	C1—C2	1.4031 (17)
F2—C8	1.3337 (16)	C1—C7	1.5011 (17)
F3—C8	1.3399 (16)	C2—C3	1.3871 (17)
O1—C7	1.2188 (16)	C2—C8	1.5114 (17)
O2—C7	1.3139 (16)	C3—C4	1.3884 (16)
O2—H2	0.851 (14)	C3—H3A	0.95
O3—N1	1.2267 (14)	C4—C5	1.3800 (17)
O4—N1	1.2271 (14)	C5—C6	1.3874 (18)
N1—C4	1.4718 (16)	C5—H5A	0.95
C1—C6	1.3950 (18)	C6—H6A	0.95
			
C7—O2—H2	108.7 (12)	C4—C5—C6	117.93 (11)
O3—N1—O4	124.05 (11)	C4—C5—H5A	121.0
O3—N1—C4	118.02 (10)	C6—C5—H5A	121.0
O4—N1—C4	117.93 (10)	C5—C6—C1	120.94 (11)
C6—C1—C2	119.61 (11)	C5—C6—H6A	119.5
C6—C1—C7	117.45 (11)	C1—C6—H6A	119.5
C2—C1—C7	122.91 (11)	O1—C7—O2	124.95 (12)
C3—C2—C1	120.02 (11)	O1—C7—C1	122.01 (12)
C3—C2—C8	117.65 (11)	O2—C7—C1	113.00 (11)
C1—C2—C8	122.18 (11)	F2—C8—F3	107.01 (11)
C2—C3—C4	118.48 (11)	F2—C8—F1	106.28 (11)
C2—C3—H3A	120.8	F3—C8—F1	106.84 (11)
C4—C3—H3A	120.8	F2—C8—C2	111.84 (11)
C5—C4—C3	122.98 (11)	F3—C8—C2	111.48 (11)
C5—C4—N1	119.23 (11)	F1—C8—C2	113.01 (11)
C3—C4—N1	117.79 (10)		
			
C6—C1—C2—C3	1.57 (18)	C4—C5—C6—C1	0.26 (19)
C7—C1—C2—C3	−176.30 (12)	C2—C1—C6—C5	−1.91 (19)
C6—C1—C2—C8	−173.81 (12)	C7—C1—C6—C5	176.08 (12)
C7—C1—C2—C8	8.32 (19)	C6—C1—C7—O1	−130.98 (14)
C1—C2—C3—C4	0.39 (18)	C2—C1—C7—O1	46.94 (19)
C8—C2—C3—C4	175.97 (11)	C6—C1—C7—O2	46.69 (16)
C2—C3—C4—C5	−2.13 (19)	C2—C1—C7—O2	−135.40 (13)
C2—C3—C4—N1	178.49 (11)	C3—C2—C8—F2	−15.31 (17)
O3—N1—C4—C5	−178.53 (12)	C1—C2—C8—F2	160.17 (12)
O4—N1—C4—C5	1.73 (17)	C3—C2—C8—F3	−135.07 (12)
O3—N1—C4—C3	0.87 (17)	C1—C2—C8—F3	40.41 (17)
O4—N1—C4—C3	−178.87 (11)	C3—C2—C8—F1	104.58 (14)
C3—C4—C5—C6	1.80 (19)	C1—C2—C8—F1	−79.94 (15)
N1—C4—C5—C6	−178.82 (11)		

### 4-Nitro-2-(trifluoromethyl)benzoic acid (I) Hydrogen-bond geometry (Å, º)

**Table:**

*D*—H···*A*	*D*—H	H···*A*	*D*···*A*	*D*—H···*A*
O2—H2···O1^i^	0.85 (1)	1.86 (2)	2.7042 (14)	175 (2)
C3—H3*A*···F3^ii^	0.95	2.47	3.3942 (15)	164

Symmetry codes: (i) −*x*+1, −*y*+1, −*z*; (ii) −*x*+3/2, *y*, *z*+1/2.

### 4-Nitro-3-(trifluoromethyl)benzoic acid (II) Crystal data

**Table:**

C_8_H_4_F_3_NO_4_	*F*(000) = 472
*M**_r_* = 235.12	*D*_x_ = 1.792 Mg m^−^^3^
Monoclinic, *P*2_1_/*n*	Mo *K*α radiation, λ = 0.71073 Å
*a* = 6.8986 (8) Å	Cell parameters from 9889 reflections
*b* = 17.240 (2) Å	θ = 2.4–30.5°
*c* = 7.6912 (9) Å	µ = 0.18 mm^−^^1^
β = 107.685 (2)°	*T* = 125 K
*V* = 871.50 (18) Å^3^	Plate, colourless
*Z* = 4	0.30 × 0.20 × 0.10 mm

### 4-Nitro-3-(trifluoromethyl)benzoic acid (II) Data collection

**Table:**

Bruker APEXII CCD diffractometer	2665 independent reflections
Radiation source: fine-focus sealed tube	2116 reflections with *I* > 2σ(*I*)
Graphite monochromator	*R*_int_ = 0.071
Detector resolution: 8.3333 pixels mm^-1^	θ_max_ = 30.5°, θ_min_ = 2.4°
φ and ω scans	*h* = −9→9
Absorption correction: multi-scan (*TWINABS*; Sheldrick, 2008a)	*k* = 0→24
*T*_min_ = 0.89, *T*_max_ = 0.98	*l* = 0→10
4385 measured reflections	

### 4-Nitro-3-(trifluoromethyl)benzoic acid (II) Refinement

**Table:**

Refinement on *F*^2^	1 restraint
Least-squares matrix: full	Hydrogen site location: mixed
*R*[*F*^2^ > 2σ(*F*^2^)] = 0.048	H atoms treated by a mixture of independent and constrained refinement
*wR*(*F*^2^) = 0.150	*w* = 1/[σ^2^(*F*_o_^2^) + (0.0909*P*)^2^ + 0.092*P*] where *P* = (*F*_o_^2^ + 2*F*_c_^2^)/3
*S* = 1.05	(Δ/σ)_max_ < 0.001
2873 reflections	Δρ_max_ = 0.50 e Å^−^^3^
150 parameters	Δρ_min_ = −0.35 e Å^−^^3^

### 4-Nitro-3-(trifluoromethyl)benzoic acid (II) Special details

**Table:**

Geometry. All esds (except the esd in the dihedral angle between two l.s. planes) are estimated using the full covariance matrix. The cell esds are taken into account individually in the estimation of esds in distances, angles and torsion angles; correlations between esds in cell parameters are only used when they are defined by crystal symmetry. An approximate (isotropic) treatment of cell esds is used for estimating esds involving l.s. planes.
Refinement. Refined as a 3-component twin. BASF 0.0961 (3) 0.0326 (2)

### 4-Nitro-3-(trifluoromethyl)benzoic acid (II) Fractional atomic coordinates and isotropic or equivalent isotropic displacement parameters (Å^2^)

**Table:**

	*x*	*y*	*z*	*U*_iso_*/*U*_eq_	
F1	0.86853 (17)	0.33150 (8)	0.38868 (15)	0.0394 (3)	
F2	0.78710 (18)	0.23188 (6)	0.51840 (16)	0.0356 (3)	
F3	0.97458 (14)	0.32129 (6)	0.68002 (13)	0.0258 (3)	
O1	0.03937 (17)	0.49961 (7)	0.22770 (15)	0.0228 (3)	
O2	0.22198 (18)	0.44236 (8)	0.06814 (16)	0.0242 (3)	
H2	0.136 (3)	0.4651 (13)	−0.015 (3)	0.029*	
O3	0.7408 (2)	0.25918 (8)	0.88052 (18)	0.0303 (3)	
O4	0.7540 (2)	0.37201 (10)	1.00889 (17)	0.0386 (4)	
N1	0.7034 (2)	0.32815 (9)	0.87729 (19)	0.0220 (3)	
C1	0.3254 (2)	0.42454 (9)	0.3879 (2)	0.0152 (3)	
C2	0.4976 (2)	0.38357 (9)	0.3829 (2)	0.0158 (3)	
H2A	0.527015	0.377515	0.27074	0.019*	
C3	0.6270 (2)	0.35142 (9)	0.5426 (2)	0.0157 (3)	
C4	0.5782 (2)	0.36199 (9)	0.7032 (2)	0.0164 (3)	
C5	0.4107 (2)	0.40416 (10)	0.7112 (2)	0.0210 (3)	
H5A	0.384027	0.411841	0.824123	0.025*	
C6	0.2823 (2)	0.43502 (10)	0.5510 (2)	0.0192 (3)	
H6A	0.16486	0.463335	0.552985	0.023*	
C7	0.1847 (2)	0.45815 (9)	0.2175 (2)	0.0168 (3)	
C8	0.8138 (2)	0.30861 (10)	0.5324 (2)	0.0216 (3)	

### 4-Nitro-3-(trifluoromethyl)benzoic acid (II) Atomic displacement parameters (Å^2^)

**Table:**

	*U*^11^	*U*^22^	*U*^33^	*U*^12^	*U*^13^	*U*^23^
F1	0.0313 (6)	0.0661 (9)	0.0253 (6)	0.0241 (6)	0.0153 (5)	0.0160 (5)
F2	0.0352 (6)	0.0242 (6)	0.0408 (7)	0.0118 (4)	0.0016 (5)	−0.0074 (5)
F3	0.0144 (4)	0.0356 (6)	0.0241 (5)	0.0043 (4)	0.0008 (4)	0.0032 (4)
O1	0.0203 (6)	0.0266 (6)	0.0190 (6)	0.0099 (5)	0.0023 (4)	−0.0002 (4)
O2	0.0243 (6)	0.0307 (7)	0.0155 (5)	0.0114 (5)	0.0027 (5)	0.0023 (5)
O3	0.0330 (7)	0.0253 (7)	0.0316 (7)	0.0080 (5)	0.0083 (6)	0.0120 (5)
O4	0.0482 (9)	0.0425 (9)	0.0169 (6)	0.0057 (7)	−0.0024 (6)	−0.0009 (6)
N1	0.0189 (6)	0.0279 (8)	0.0186 (7)	0.0042 (5)	0.0050 (5)	0.0076 (5)
C1	0.0138 (6)	0.0145 (7)	0.0156 (7)	0.0004 (5)	0.0018 (5)	0.0012 (5)
C2	0.0151 (6)	0.0165 (7)	0.0151 (7)	0.0011 (5)	0.0034 (5)	0.0005 (5)
C3	0.0141 (6)	0.0148 (7)	0.0167 (7)	0.0011 (5)	0.0022 (5)	0.0005 (5)
C4	0.0160 (7)	0.0154 (7)	0.0155 (7)	0.0002 (5)	0.0016 (5)	0.0029 (5)
C5	0.0208 (7)	0.0262 (9)	0.0173 (7)	0.0035 (6)	0.0076 (6)	0.0028 (6)
C6	0.0154 (7)	0.0217 (8)	0.0198 (7)	0.0037 (6)	0.0044 (6)	0.0012 (6)
C7	0.0168 (6)	0.0159 (7)	0.0161 (7)	0.0018 (5)	0.0028 (5)	−0.0002 (5)
C8	0.0197 (7)	0.0255 (8)	0.0180 (7)	0.0073 (6)	0.0035 (6)	0.0033 (6)

### 4-Nitro-3-(trifluoromethyl)benzoic acid (II) Geometric parameters (Å, º)

**Table:**

F1—C8	1.332 (2)	C1—C2	1.393 (2)
F2—C8	1.335 (2)	C1—C7	1.491 (2)
F3—C8	1.3424 (18)	C2—C3	1.395 (2)
O1—C7	1.2528 (18)	C2—H2A	0.95
O2—C7	1.281 (2)	C3—C4	1.387 (2)
O2—H2	0.826 (16)	C3—C8	1.508 (2)
O3—N1	1.2154 (19)	C4—C5	1.383 (2)
O4—N1	1.226 (2)	C5—C6	1.387 (2)
N1—C4	1.4751 (19)	C5—H5A	0.95
C1—C6	1.386 (2)	C6—H6A	0.95
			
C7—O2—H2	107.5 (16)	C4—C5—C6	118.62 (15)
O3—N1—O4	125.51 (15)	C4—C5—H5A	120.7
O3—N1—C4	117.88 (14)	C6—C5—H5A	120.7
O4—N1—C4	116.54 (15)	C1—C6—C5	119.98 (15)
C6—C1—C2	120.69 (13)	C1—C6—H6A	120.0
C6—C1—C7	118.92 (14)	C5—C6—H6A	120.0
C2—C1—C7	120.39 (14)	O1—C7—O2	124.04 (14)
C1—C2—C3	120.00 (14)	O1—C7—C1	119.12 (14)
C1—C2—H2A	120.0	O2—C7—C1	116.84 (13)
C3—C2—H2A	120.0	F1—C8—F2	107.05 (14)
C4—C3—C2	117.95 (14)	F1—C8—F3	106.48 (14)
C4—C3—C8	123.56 (13)	F2—C8—F3	106.73 (13)
C2—C3—C8	118.48 (14)	F1—C8—C3	111.13 (13)
C5—C4—C3	122.73 (14)	F2—C8—C3	112.97 (14)
C5—C4—N1	115.73 (14)	F3—C8—C3	112.10 (14)
C3—C4—N1	121.53 (14)		
			
C6—C1—C2—C3	1.1 (2)	C2—C1—C6—C5	−0.4 (2)
C7—C1—C2—C3	−179.15 (14)	C7—C1—C6—C5	179.87 (15)
C1—C2—C3—C4	−0.2 (2)	C4—C5—C6—C1	−1.2 (2)
C1—C2—C3—C8	−179.01 (14)	C6—C1—C7—O1	5.0 (2)
C2—C3—C4—C5	−1.5 (2)	C2—C1—C7—O1	−174.71 (15)
C8—C3—C4—C5	177.26 (15)	C6—C1—C7—O2	−175.09 (15)
C2—C3—C4—N1	178.27 (14)	C2—C1—C7—O2	5.2 (2)
C8—C3—C4—N1	−3.0 (2)	C4—C3—C8—F1	−155.52 (15)
O3—N1—C4—C5	127.62 (17)	C2—C3—C8—F1	23.2 (2)
O4—N1—C4—C5	−49.6 (2)	C4—C3—C8—F2	84.14 (19)
O3—N1—C4—C3	−52.1 (2)	C2—C3—C8—F2	−97.15 (17)
O4—N1—C4—C3	130.66 (17)	C4—C3—C8—F3	−36.5 (2)
C3—C4—C5—C6	2.2 (2)	C2—C3—C8—F3	142.23 (14)
N1—C4—C5—C6	−177.56 (14)		

### 4-Nitro-3-(trifluoromethyl)benzoic acid (II) Hydrogen-bond geometry (Å, º)

**Table:**

*D*—H···*A*	*D*—H	H···*A*	*D*···*A*	*D*—H···*A*
O2—H2···O1^i^	0.83 (2)	1.82 (2)	2.6337 (16)	168 (2)
C5—H5*A*···O2^ii^	0.95	2.51	3.440 (2)	165

Symmetry codes: (i) −*x*, −*y*+1, −*z*; (ii) *x*, *y*, *z*+1.
